# Burden of leptospirosis in Brazil in the last decade

**DOI:** 10.11606/s1518-8787.2024058005859

**Published:** 2024-12-16

**Authors:** Gisele de Paula Linhares, Tiago Zequinao, Gustavo Martini Buso, June Alisson Westarb Cruz, Felipe Francisco Tuon

**Affiliations:** I Pontifícia Universidade Católica do Paraná. Laboratório de Doenças Infecciosas Emergentes. Curitiba, PR, Brasil; II Pontifícia Universidade Católica do Paraná. Escola de Negócios. Curitiba, PR, Brasil

**Keywords:** Leptospirosis, Incidence, Economic Data, Ecological Study, Healthy System

## Abstract

To correlate the incidence of leptospirosis with sociodemographic data in the Brazilian Unified Health System from 2011 to 2022.

This ecological study used national health and economic secondary data sources. Secondary analyses summarized the scenario of disease-related hospitalizations among federative units. In total, two analyses were conducted: variable description for relationship analysis and a secondary analysis with population health and sanitation indicators and economic indicators from the *Instituto Brasileiro de Geografia e Estatística* (IBGE - Brazilian Institute of Geography and Statistics). The statistical analysis following this framework summarized raw data by year-month-federative unit. A time series regression was conducted, comparing the time variable with other national-level variables. Then, several simple linear regressions were performed.

Linear regressions show the relationship between the reduction in cases and improved access to treated water and sewage collection, whereas an increase in *per capita* income seems to be inversely related to leptospirosis incidence. Geospatial distribution shows higher incidence in the Brazilian South and Southeast. Disease lethality varied over time but without significant change during the period. The average treatment cost remained constant over the years, despite its complexity.

Leptospirosis incidence in Brazil from 2011 to 2021 decreased and was associated with improvements in socioeconomic conditions despite no changes in lethality.

## INTRODUCTION

 Described for the first time in 1886 by Adolf Weil, leptospirosis has been a serious disease with significant issues related to its incidence for decades, potentially leading to multiple organ failure and a high mortality rate. Estimates suggest that over a million new cases and more than 58,000 deaths occur worldwide annually ^2023^ . 

 From 2010 to 2020, Brazil confirmed 39,270 cases of leptospirosis, 68.7% of which required hospitalization ^2021^ . This indicates not only the occurrence of moderate and severe cases but also the underreporting of the disease in its early phase. A study in Brazil showed varying mortality rates: Acre, 1.13%; Espírito Santo, 4.89%; Rio Grande do Sul, 5.82%; Santa Catarina, 3.91%; Paraná, 11.40%; Pernambuco, 12.13%; São Paulo, 12.23%; Pará, 12.58%; Bahia, 14.34%; and Rio de Janeiro, 18.74%, representing 9.65% of leptospirosis-related deaths from 2000 to 2017 ^2020^ . These data, as alarming as they may be, fail to stimulate interest in the pharmaceutical industry, especially in vaccine production, diagnostic methods, or medications beyond traditional antibiotics. 

 In this structural historical context, leptospirosis, an infectious disease caused by the bacterium *Leptospira* , directly impacts people’s productive lives and the economic situation due to the high hospitalization costs ^2018^ . It stands out as a disease with a high prevalence of treatment within the Brazilian Unified Health System (SUS), with more than 68,000 notifications in Brazil from 2000 to 2016 ^2020^ . 

This study aims to identify correlations between leptospirosis incidence, infrastructure factors, and sociodemographic data from the last decade in Brazil.

## METHODS

### Study Design

This descriptive, exploratory and ecological study was conducted with secondary health and national economic data to relate the incidence of leptospirosis to infrastructure and sanitation access in Brazil from 2011 to 2021. A secondary analysis was performed to overview leptospirosis hospitalizations across the Brazilian federal units.

 With a population of approximately 215 million people spread over a territorial area of 8.5 million km ^2021^ , Brazil, a country with a tropical climate, serves as the backdrop for several diseases and ailments ^2019^ . Access to healthcare in Brazil has been established as a universal right by its people via their constitutional power, with healthcare becoming a constitutional right for the entire population since its 1988 Federal Constitution, a right that is guaranteed and operationalized by SUS. 

### Description of Variables for Relationship Analysis

 Data were collected from the Saneamento Brasil Panel of the Trata Brasil Institute ^2019^ . This database includes municipal-level sanitation data from 839 municipalities and metropolitan regions in Brazil with populations over 50,000 inhabitants. Data were organized by federal unit and year, excluding 2022 due to unavailable sanitation data. The following variables were analyzed by time-series regression and linear regression: time (in years), cases (representing the total number of leptospirosis-related hospitalizations), cases per 100,000 inhabitants (hospitalizations per population), population without access to treated water (percentage of people without access to potable water in a state), population without sewage collection (percentage of people without sewage collection and treatment), *per capita* income with sanitation, *per capita* income without sanitation (representing the average income in Brazilian reais for individuals with or without sewage collection in their residences). 

### Description of Secondary Analysis Variables

 Data from the secondary analysis were collected from the TAbNet/DataSUS system ^2020^ , a platform developed by the Brazilian Ministry of Health containing health population indicators and sanitation data. Economic indicators were collected from the *Instituto Brasileiro de Geografia e Estatística* (IBGE - Brazilian Institute of Geography and Statistics) ^2011^ . 

The following variables were analyzed: number of leptospirosis hospitalizations, total hospitalizations, average length of stay (the average number of days during hospitalization up to discharge), number of deaths from the disease, lethality rate (the ratio of number of deaths in relation to the total hospitalizations for the disease), and average revenue (sum received by the hospital for each SUS-performed hospitalization). Monthly and accumulated data from the National Consumer Price Index for Health Services (IPCA) were used for monetary value adjustments, with a conversion rate of R$ 5.05 (corresponding to the US dollar exchange rate on the analysis date).

### Statistical Analysis

Raw data were summarized in the pre-analytical phase, in which months and federal units were represented as single data points to depict national data by year. For the relationship analysis, a time series regression was initially conducted, comparing the time variable with other national variables. Subsequently, various simple linear regressions were performed, with cases per 100,000 inhabitants as the dependent variable and infrastructure and sanitation access data from the federal units as independent variables. A linear regression model was used for time series analysis with Prais-Winsten estimations, the statistical significance of which was defined as p-value < 0.05. A simple linear regression was performed for variables unrelated with time. For the secondary analysis, data were shown in line and bar graphs to illustrate national data. Heatmaps using the geographical map of Brazil aimed to show the evolution of variables by federal unit. Data tabulation, table and graph development, heatmap creation, and regression analyses were conducted using Stata 18, Adobe Illustrator 2021, and SPSS v.21.0, respectively.

## RESULTS

### Temporal Series

**Figure 1.**
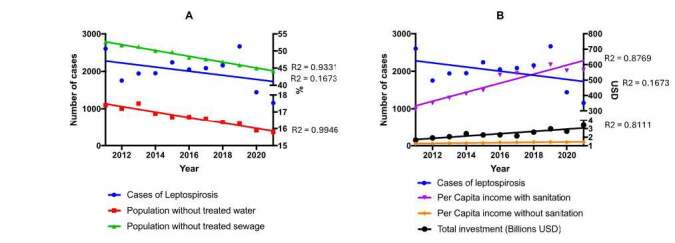
Time series analysis of leptospirosis cases, sanitation, income, and public sanitation investment in Brazil.

Note: A: number of cases × percentage × year. B: number of cases × Brazilian reais × year. The counting in billions is expressed at the bottom of the z-axis. Markings represent the disper sion of the points and the regression line for each variable, with their respective R² values.

 The incidence of leptospirosis in Brazil varied from 16.70 to 39.26 cases per 100,000 inhabitants from 2011 to 2021. Cases have considerable dispersion and a weak relationship (R² = 0.1673) with the time series ( Figure 1 ). Moreover, the population without access to treated water and the population without sewage collection significantly decreased over the years (percentage difference = -9.20%; -15.97%, and R² = 0.9331; R² = 0.9946). Figure 1 in the supplementary material ^a^ details these data (supplementary Table H). 

 The *per capita* income of both the population with and without sanitation, despite the average difference of R$ 398.11, showed a positive relationship with the time series and increase over the years (percentage difference = 81.91% for both and R² = 0.8769; R² = 0.8111). Similarly, total investment showed a strong relationship with the time series and more than doubled during the period (percentage difference = 106.21%; R² = 0.8111). The variables of untreated sewage, the index of treated sewage relative to consumed water, total hospitalizations, and total deaths from waterborne diseases showed no significant p values. 

### Linear Regression

**Figure 2.**
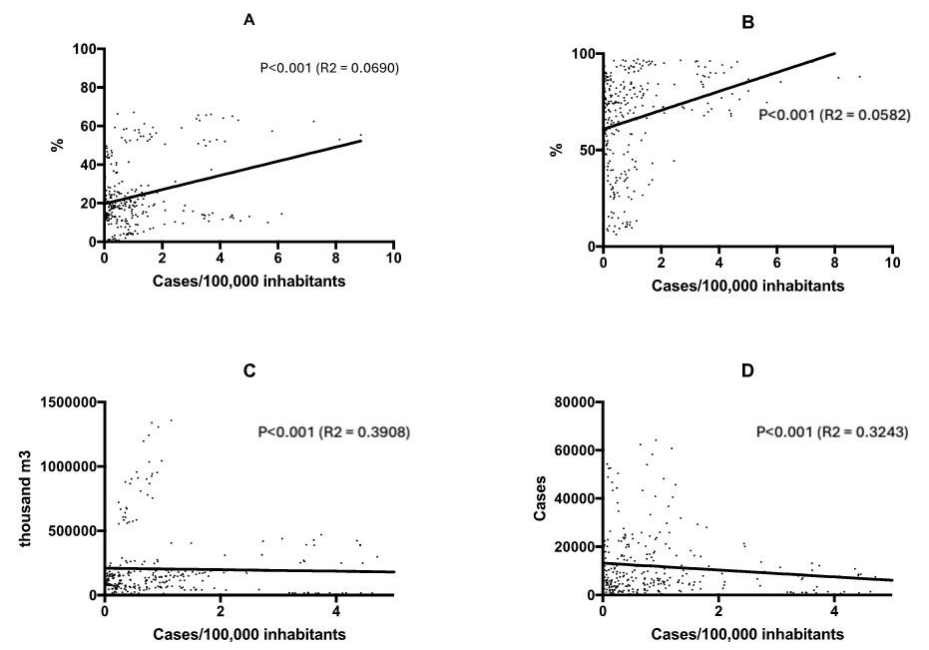
Simple regression of the number of cases/100,000 inhabitants and sanitation variables.

Note: A: number of cases/100,000 inhabitants by population without access to treated water; B: number of cases/100,000 inhabitants by population without sewage collection; C: number of cases/100,000 inhabitants by untreated sewage (thousand m³); D: number of cases/100,000 inhabitants by total hospitalizations for waterborne diseases. Markings represent the dispersion of the points and the regression line for each variable, with their respective p and R² values.

 The simple linear regression analysis observed that sanitation variables have a low relationship with the number of leptospirosis cases per 100,000 inhabitants. of the variables that showed significant p values, untreated sewage seems to best explain the variation in the number of cases per 100,000 inhabitants, with an R² = 0.3908 ( Figure 2 ). The population without access to treated water, those without sewage collection, and total hospitalizations for waterborne diseases, although significant, had irrelevant R² values. Sewage treatment index relative to consumed water, deaths from waterborne diseases, income of the population with and without sanitation, and total investment showed no statistical significance. 

### Distribution by Regions

 The following indicators represent the number of hospitalizations, average length of hospital stay, lethality rate, average cost per hospitalization, and total reimbursement to SUS regarding leptospirosis cases in Brazil. The average number of hospitalizations for leptospirosis in Brazil from 2011 to 2022 totaled 1,999 cases per year. The number of cases during the initial period showed stability, peaking in 2019, followed by a sharp decrease in 2020 and 2021 (supplementary material ^a^ ). 

**Figure 3.**
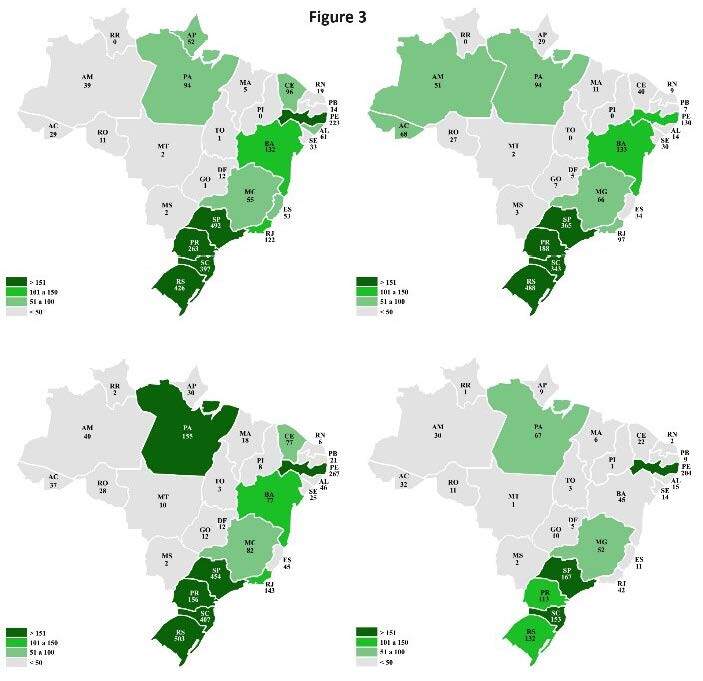
Distribution of hospitalizations in Brazil per state in 2011, 2015, 2019, and 2021.

 A detailed analysis with temporal cuts for 2011, 2015, 2019, and 2021 found that the Brazilian South and Southeast had the highest prevalence of hospitalizations due to the disease over the years, whereas the Midwest had the lowest numbers. The states with the highest average hospitalizations were Rio Grande do Sul (387), São Paulo (370), Santa Catarina (325), Pernambuco (206), and Paraná (180) when compared to the national average of 81 cases per state. On the other hand, Roraima (0.75), Tocantins (1.75), Piauí (2.25), Mato Grosso do Sul (2.25), and Mato Grosso (3.75) had the lowest number of hospitalization cases ( Figure 3 ). 

Data source: TabNet/Datasus system, 2022.

Maps: 2011: upper left; 2015: upper right; 2019: lower left; 2021: lower right.

Among the main variations over time, the following states showed a sustained decrease in cases: Paraná, with reductions of -29%, -17%, and -28% (2011–2015, 2015–2019, 2019–2021); Rio Grande do Norte, -53%, -33%, -67%; and Sergipe, -9%, -17%, -44%. Meanwhile, sustained increases occurred in Goiás, 600% and 71% (2011–2015, 2015–2019); Rondônia, 146% and 4%; Maranhão, 120% and 64%; Minas Gerais, 20% and 24%; and Rio Grande do Sul, 15% and 3%.

A global reduction in cases is evident in 2021. Among the 26 Brazilian states, in addition to the Federal District, only Mato Grosso do Sul and Tocantins showed no reduction in the number of hospitalizations from 2019 to2021. However, these states have low case registration throughout the historical series.

### Hospital Length-of-Stay

**Figure 4.**
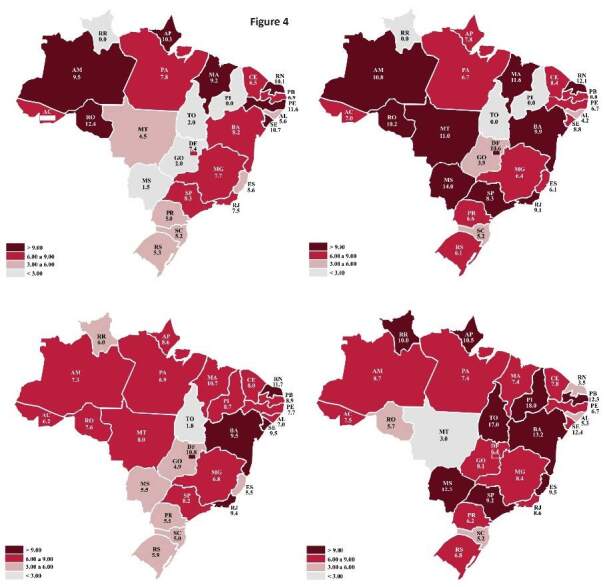
Average length of stay per state in Brazil in 2011, 2015, 2019, and 2021.

 The average length of hospital stay throughout the study period totaled 8.18 days. Nationwide data initially showed stability, peaking at 9.43 days in 2018 and tending to fall thereafter (supplementary material ^a^ ). A detailed analysis by Brazilian region shows that the Northeast had the highest average length of hospital stay during the analyzed period, whereas the South had the lowest numbers. The following states had the highest average length of stay: Piauí (13.4 days), Sergipe (10.3 days), Bahia (10.2 days), Maranhão (9.8 days), and Paraíba (9.5 days), when compared to the national average (8.18 days). On the other hand, Goiás (4.8 days), Santa Catarina (5.1 days), Alagoas (5.6 days), Paraná (5.8 days), and Rio Grande do Sul (6.0 days) had the lowest average hospital stay among the states (Figure 4). Regarding variations by state over the years, note that Rondônia had a consistent reduction of −18%, −26%, and −25%. Conversely, Goiás showed an increase in the average length of stay inn all periods: 97, 24, and 66%. Other states showed varying behavior over the years. 

Data source: TabNet/Datasus system, 2022.

Maps: 2011: upper left; 2015: upper right; 2019: lower left; 2021: lower right.

### Mortality

 The nationwide case fatality rate (CFR) averaged 4.51% during the period. The indicator showed a declining trend over time, with two evident peaks in 2018 at 5.30% and a higher one in 2020 at 6.58% (supplementary material ^a^ ). 

**Figure 5.**
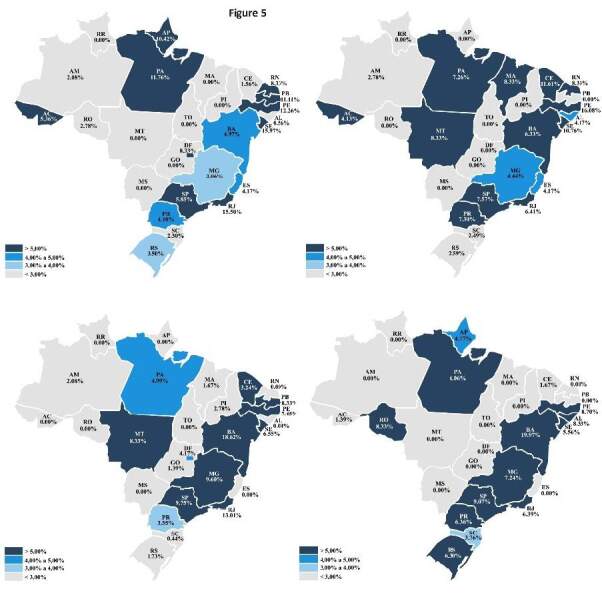
Fatality rate per state in Brazil in 2011, 2015, 2019, and 2021.

 Notably, the Brazilian Northeast and Southeast had the highest CFR in the analyzed period, whereas the Midwest and North, the lowest numbers. The states with the highest CFR were Bahia (12.5%), Pernambuco (11.2%), Rio de Janeiro (10.3%), Sergipe (9.7%), and São Paulo (8.1%). In some cases, these rates totaled more than double the national average (4.51%). On the other hand, Mato Grosso, Tocantins, Roraima, Goiás, and Piauí had an average CFR close to zero, which is correlated with the extremely low number of reported cases in these states. Of the states with an average of more than 30 hospitalizations per year, the following showed the lowest mortality rates: Amazonas (1.7%), Espírito Santo (2.1%), and Santa Catarina (2.3%) ( Figure 5 ). 

Data source: TabNet/Datasus system, 2022.

Maps: 2011: upper left; 2015: upper right; 2019: lower left; 2021: lower right.

Noteworthy variations in the CFR over the period include Sergipe, which had a continuous reduction of −33%, −39%, and a subsequent increase of 15%. Meanwhile, Bahia showed a continuous increase in CFR: 27%, 194%, and 7%. Other states showed volatile behavior over the years, especially those with a low number of hospitalizations. In these cases, a single patient who passed away could result in a significant fluctuation in the indicator.

### Average Revenue and Inflation in the Health Sector

 The supplementary material ^a^ describes the average sum paid to hospitals for leptospirosis admissions. Prices ranged from $108.60 to $209.88 during the period, averaging $158.95. The period showed a trend of increasing prices, with a cumulative variation of 80.0%. An increase in variation occurs up to 2014, followed by a decrease from 2015 to 2017 and an increasing trend from 2019 onward. The most significant variation peaks occurred in 2018 (33%) and 2019 (with a −10% variation). 

 The IPCA accumulated a 95.6% variation during the studied period (supplementary material), indicating that healthcare service prices almost doubled during this time. 2013 showed an increase in prices, with a visible plateau from 2015 to 2017, followed by a decline from 2018 to 2021. When compared, the variations in average revenue and the IPCA show discrepancies in various periods both individually and when accumulated. Mostly, inflation is above the variation in average revenue over time. The period from 2016 to 2019 shows a noticeable gap in its data (supplementary material ^a^ ). In only four years, the average revenue adjustment exceeded the IPCA, whereas such variation was lower in other years, including negative values in 2016, 2017, 2019, and 2021. 

 In general, cost variation (represented by inflation) and revenue clearly diverge from each other. The supplementary material ^a^ (supplementary Figure G) shows the scenario in which financial transfers to hospitals are adjusted according to the IPCA. The nominal value refers to the value of the average revenue per leptospirosis admission, whereas the real value considers the discussed adjustment. Note the considerable gap in values and the progressive increases over the years. 

## DISCUSSION

 Leptospirosis is an infection that is directly related to the population of urban rats and climatic conditions ^2020^ . Its occurrence requires the source of infection (the rat), the pathogen ( *Leptospira* ), and water contaminated with urine. Thus, three factors are necessary for the disease to spread. In Brazil, long-standing sanitary conditions, along with a high rat population and the presence of the bacteria, meet the criteria for a high incidence of leptospirosis. Brazil has an intermediate to high incidence, whereas highest incidences occur in sub-Saharan Africa and Southeast Asia, excluding Australia and New Zealand ^2015^ . 

 Given this landscape, it is possible to discuss the data in this study. Although the incidence varied from 2011 to 2021, it tends toward reduction. Due to the absence of leptospirosis vaccine, the justifications for the reduction in cases include improving the population’s quality of life, socio-economic factors, and sanitation conditions. The analysis of IBGE data shows an increase in *per capita* income, health investment, and access to treated water and a decrease in the population without access to sewage collection. The combination of these factors explains the reduction in the number of cases. However, as this is an ecological study, it is impossible to determine which variable has the greatest impact ^2017^ . Although we can identify correlations or regressions to determine the highest variation in one of these variables, small changes can sometimes have an impact. For example, a significant increase in income is insufficient without improving access to treated water or sewage collection. Similarly, only an increase in health sector investment could explain a reduction in leptospirosis incidence. 

 The linear regression between the incidence of leptospirosis cases from 2011 to 2021 shows a clear relationship between the reduction in cases and a decrease in the population without access to treated water and sewage collection and an inverse relationship between an increase in *per capita* income and leptospirosis incidence. These linear regressions confirm the hypotheses in Table 1 and Table 2 . A study conducted in Rio Grande do Sul was unable to relate the number of leptospirosis cases to sewage collection but found this factor to be a risk for lethality ^2023^ . Rather than an isolated factor associated with mortality, it seems to offer a risk factor for acquiring the disease. Perhaps the bias in interpretation stems from people in these conditions having poorer access to good healthcare, those with low income and less awareness of the severity of the disease delay seeking prompt treatment, or even that treatment and life support are used ineffectively ^2008^ . 

**Table 1.** Leptospirosis cases, sanitation, income, and public investment in sanitation in Brazil.

Year	Cases of leptospirosis	Cases/100,000 inhabitants	Population without treated water	Population without sewage	Per capita income sanitation (USD)	Per capita income without sanitation (USD)	Investment (billions of USD)
2011	2,605	39.26	17.40%	52.60%	311.30	52.94	1.66
2012	1,757	27.38	17.20%	51.70%	350.21	59.56	1.93
2013	1,943	29.05	17.50%	51.30%	384.10	65.32	2.07
2014	1,950	35.06	16.90%	50.10%	410.35	69.79	2.42
2015	2,241	35.07	16.70%	49.70%	433.68	73.76	2.26
2016	2,050	28.61	16.70%	48.10%	533.94	90.81	2.28
2017	2,086	29.31	16.60%	47.60%	544.14	92.54	2.16
2018	2,162	32.26	16.40%	46.90%	583.58	99.25	2.59
2019	2,666	35.99	16.30%	45.90%	599.62	101.98	2.99
2020	1,444	19.81	15.90%	45.00%	560.68	95.36	2.70
2021	1,159	16.70	15.80%	44.20%	566.29	96.31	3.42

USD: American dollar.

**Table 2.** Linear regression model for time series analysis with the Prais-Winsten for leptospirosis cases, sanitation, income, and public investment in sanitation in Brazil.

Variable	Coefficient	95% confidence interval	Significance
Cases of leptospirosis	-62,351	[164,427 to 39,723]	No
Population without treated water	-0.163	[-0.191 to -0.135]	Yes
Population without treated sewage	0.852	[-0.882 to -0.8217]	Yes
*Per Capita* income with sanitation	27,254	[14,635 to 39,873]	Yes
*Per Capita* income without sanitation	4,635	[2,489 to 6,781]	Yes
Total investment (Billions USD)	0.132	[0.088 to 0.176]	Yes

USD: American dollar.

 The geospatial evaluation of leptospirosis in Brazil draws attention to a higher number of cases in its South and Southeast ^2023^ , the most populous areas in the country with the highest socio-economic development ^2022^ . An explanation for this phenomenon refers to the local metropolises that house a higher rat population, making disease transmission easier. We can confirm this with the high incidence in some Northeastern states, such as Pernambuco and Bahia, which have also experienced an increase in large cities. The Brazilian North and Midwest consist of large rural areas and fewer metropolises, which reduce the urban rat population and favor wild rats, which are associated with hantavirus rather than with leptospirosis ^2023^ . 

 It is interesting to evaluate the data on the average length of hospital stay for leptospirosis across states and regions of Brazil. The highest rates of hospital stay occur in the North, Midwest, and Northeast, exactly the regions with lower leptospirosis incidence. Possible explanations for this situation include low incidences, delayed recognition of the disease ^2023^ , and late diagnoses and treatment. Another possibility is the difficult access of the population to the healthcare system, resulting in treatment only for more severe cases with prolonged hospitalization ^2022^ . Differences in *Leptospira* serovars may be associated with different clinical presentations and severity ^2014^ . 

 The lethality of leptospirosis ranged from 3.11 to 6.48%, with the last two years of the analysis having the lowest values. Evaluating the lethality map in Brazil shows an asynchronous relationship with the length of hospitalization as the South, Southeast, and Northeast show similar rates. Multiple risk factors are associated with leptospirosis lethality. Characteristics of the bacteria, patient factors, and healthcare resources for managing severe cases are independent factors ^2018^ . Regarding the bacteria, *Leptospira* has several variants, called serovars ^2023^ . These variants have different genes associated with virulence factors ^2022^ . The most well-known are Icterohaemorrhagiae and Copenhageni, although more than 10 serovars exist, Each of which shows variable virulence and mortality and different behaviors depending on factors such as rainfall, soil pH, temperature, and vector animals other than rats ^2019^ . Therefore, one possibility to explain the variability in lethality is related to serovars. 

 Lethality in leptospirosis can also be associated with the patient’s genetic condition ^2021^ . Patients with polymorphisms in cytokine receptors may have more severe or milder cases due to variable immune responses. Lethality can also be related to the life support capacity of the hospital center. Severe leptospirosis patients may require intensive care and therapy, including invasive mechanical ventilation, hemodialysis for renal failure treatment, or metabolic disorders, as well as routine laboratory tests with rapid results for prompt decision making ^2020^^–^^2011^ . The average cost of treating leptospirosis remained the same over 10 years, even when adjusted for the IPCA, evincing no increase in reimbursement for this disease. The treatment of severe leptospirosis requires prolonged hospitalization, intensive therapy, mechanical ventilation, hemodialysis, as well as intravenous antibiotics and even blood products ^2014^^,^^2010^ . 

 This descriptive and analytical ecological study described aggregated data. Therefore, it is unable to extrapolate the results to individuals or evaluate risk factors. The main objective of ecological studies is to evaluate aggregated data to generate hypotheses about outcome variables. Confirming these hypotheses requires other types of studies. In addition to the inherent ecological bias in this type of study, this research also highlights the fragility of the data acquired from Datasus systems. Notification forms can incur in errors during filling out and diagnosis that can compromise the faithful interpretation of the data in this study. This research ignored rainfall by region since it is directly related to an increase in the number of cases ^2019^ . However, the diagnosis of leptospirosis depends on laboratory tests, which are often inaccessible in many Brazilian municipalities. Health services with fewer epidemiological resources may also fail to record this notifiable disease. 

Thus, the incidence of leptospirosis in Brazil from 2011 to 2021 decreased and showed a relationship with the increase in sewage collection, the availability of treated water, and family income. The average cost of treating leptospirosis and its lethality rate remained the same in Brazil. Finally, the highest incidence of leptospirosis occurs in Southern and Southeastern states, but the length of hospitalization is shorter in these regions when compared to the North, Northeast, and Midwest.

Considering these conclusions, Brazil should improve social conditions, which would further reduce the incidence of leptospirosis. Additionally, two important aspects that need immediate reconsideration refer to training healthcare teams on diagnosis and treatment and increasing financial investment in life support for these patients.

## Footnotes

1 Available from: https://docs.google.com/document/d/1ngL3ikjarenXK5_9nohf0ukNE66sSQbP/edit?usp=sharing&ouid=103913096382451928465&rtpof=true&sd=true
