# Supplementary material for: Burden of leptospirosis in Brazil in the last decade
Source: Rev Saude Publica. 2024 Dec 16;58:53. doi: 10.11606/s1518-8787.2024058005859 (PMC11655057; doi:10.11606/s1518-8787.2024058005859)
Supplement: Supplementary file 1 [file 1518-8787-rsp-58-53-suppl1.pdf]

Supplementary material

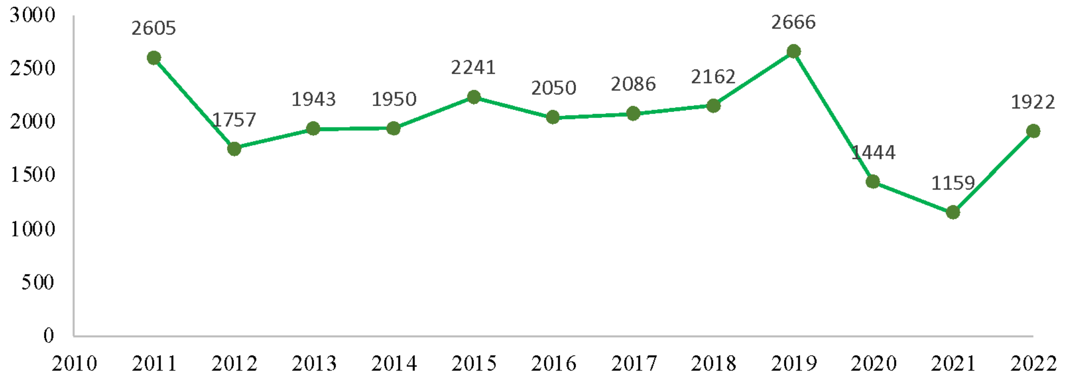

Figure A. Count of hospitalizations in Brazil from 2011 to 2022. Data source: TabNet/DataSUS System, 2023. The vertical axis represents the number of cases, and the horizontal axis represents the years of analysis.

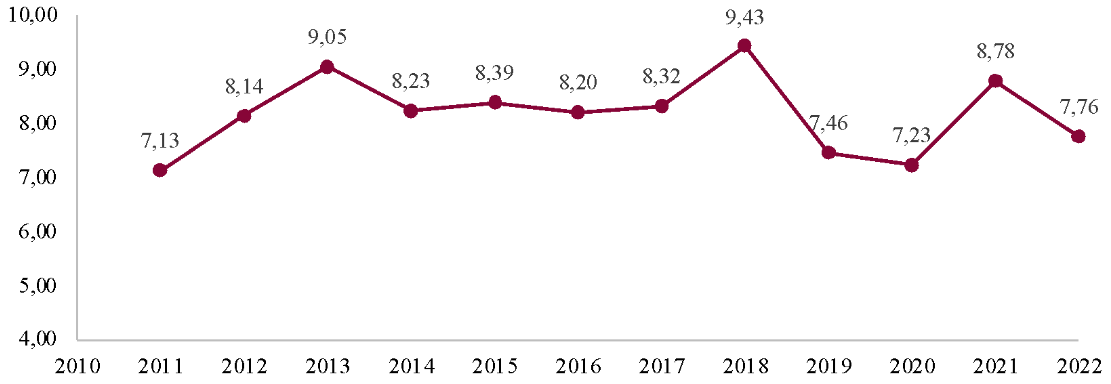

Figure B. Average length of hospital stay in Brazil from 2011 to 2022. Data source: TabNet/DataSUS system, 2023. ("The values are in Brazilian numerals; to convert to English numbers, replace the commas with periods.")

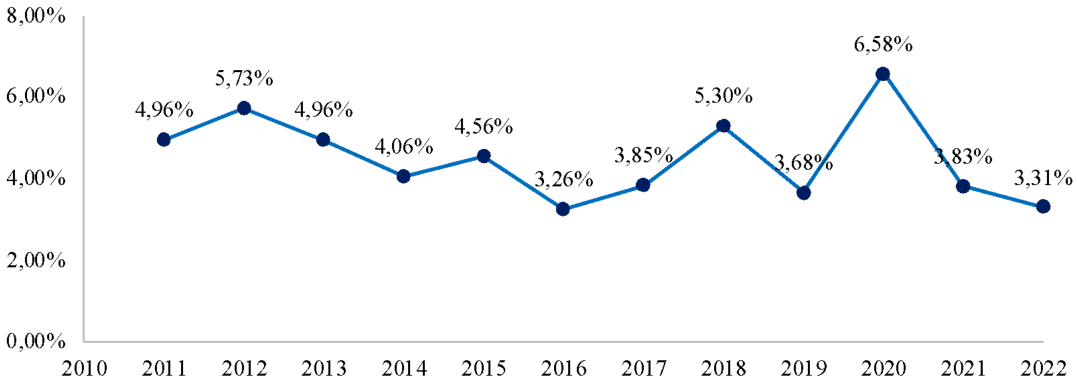

<u>Figure C – Fatality Rate in Brazil</u> from 2011 to 2022. Source: TabNet System/DataSUS, 2023. ("The values are in Brazilian numerals; to convert to English numbers, replace the commas with periods.")

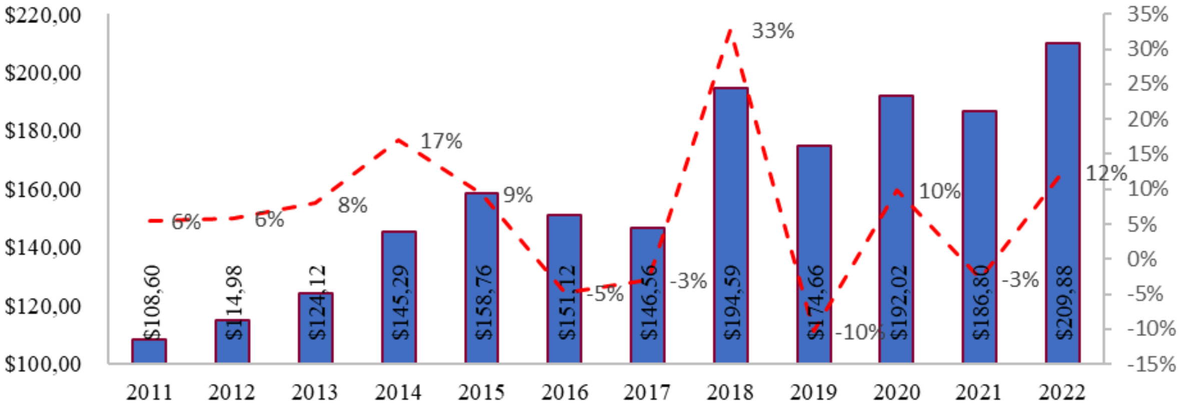

<u>Figure D. Average Ticket and Variation in Average Ticket</u> per Hospitalization in Brazil from 2011 to 2022. Source: TabNet/DataSUS System, 2023.

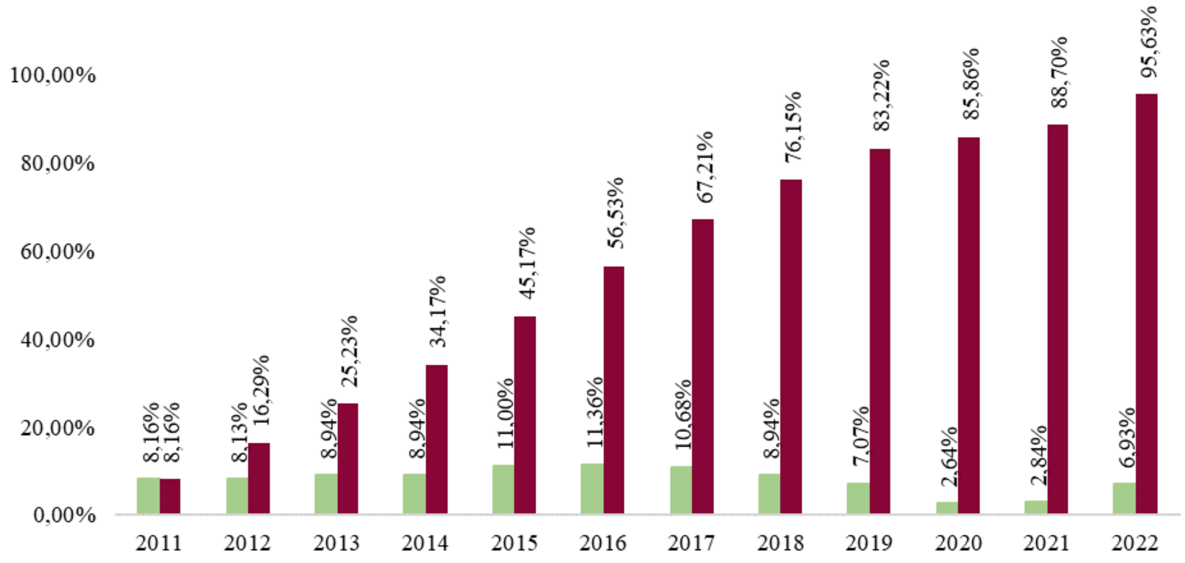

<u>Figure E. Consumer Price Index Health</u> Yearly and Accumulated in Brazil from 2011 to 2022.

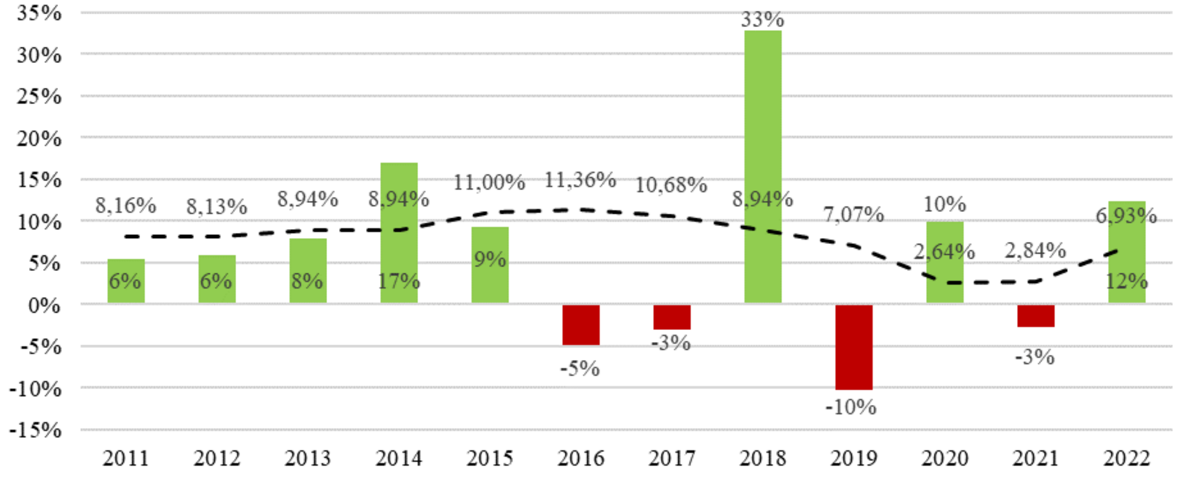

<u>Figure F. Annual Average Ticket Price Variation</u> and Annual Health CPI Variation. Columns: Annual Ticket Price Variation. Dashed Line: Annual Health CPI Variation. Source: The Author, 2023.

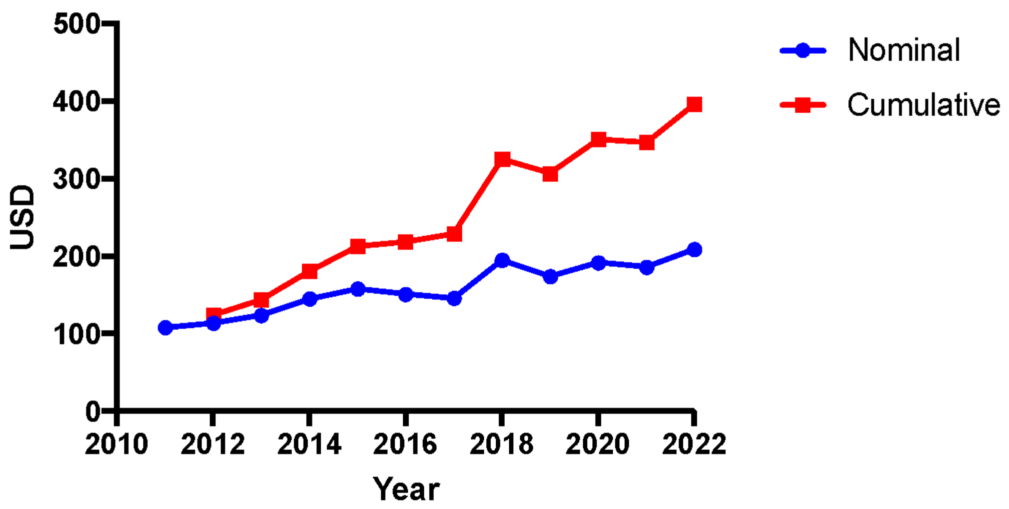

Figure G. **Real and nominal values accumulated** (average ticket prices) in Brazil from 2011 to 2022.

Year	Cases of leptospirosis	Cases/ 100.000 inhabitants	Population without treated water	Population without sewage	per capita income sanitation (USD)	per capita income without sanitation (USD)	Investment (billions of USD)
2011	2605	39.26	17.40%	52.60%	311.30	52.94	1.66
2012	1757	27.38	17.20%	51.70%	350.21	59.56	1.93
2013	1943	29.05	17.50%	51.30%	384.10	65.32	2.07
2014	1950	35.06	16.90%	50.10%	410.35	69.79	2.42
2015	2241	35.07	16.70%	49.70%	433.68	73.76	2.26
2016	2050	28.61	16.70%	48.10%	533.94	90.81	2.28
2017	2086	29.31	16.60%	47.60%	544.14	92.54	2.16
2018	2162	32.26	16.40%	46.90%	583.58	99.25	2.59
2019	2666	35.99	16.30%	45.90%	599.62	101.98	2.99
2020	1444	19.81	15.90%	45.00%	560.68	95.36	2.70
2021	1159	16.70	15.80%	44.20%	566.29	96.31	3.42

USD – American dollar

Table H. Leptospirosis cases, sanitation, income, and public investment in sanitation in Brazil
